# Stigma perception and determinants among patients with type 2 diabetes mellitus in Iraq

**DOI:** 10.1186/s42506-023-00145-5

**Published:** 2023-11-29

**Authors:** Taqi M. J. Taher, Hussein A. Ahmed, Ali A. Abutiheen, Shaymaa A. Alfadhul, Hasanain F. Ghazi

**Affiliations:** 1 Family and Community Medicine Department, College of Medicine, Wasit University, Wasit, Iraq; 2 Family and Community Medicine Department, Faculty of Medicine, University of Kufa, Kufa, Iraq; 3 Family and Community Medicine Department, College of Medicine, University of Kerbala, Kerbala, Iraq; 4 College of Nursing, Al-Bayan University, Baghdad, Iraq

**Keywords:** Diabetes, Type 2 DM, Stigma, Diabetic stigma, Iraq

## Abstract

**Background and purpose:**

Diabetes mellitus (DM) is a leading cause of death worldwide. Stigma is a sign of social disgrace occurring within public relations, and it is linked with many health conditions including diabetes. Stigma could worsen the disease course, reduce treatment adherence, and affect the quality of life of diabetic patients. The objective of this study was to assess the magnitude of diabetic stigma among patients with type 2 DM.

**Methods:**

In this analytic cross-sectional study, data collection was performed from June 1, 2022, until November 1, 2022, et al.-Najaf City, Iraq. A consecutive sample of 429 patients with type 2 DM was interviewed using the Arabic version of the type 2 Diabetes Stigma Assessment Scale (DSAS-2), which is a validated tool. The total diabetic stigma score, treated differently score, self-stigma score, and blame and judgment score were estimated.

**Results:**

The mean age of the sample was 56.6 years, and males represented 61.8% of them. The total diabetic stigma score mean was 51.72. The question regarding people's judgment of food choices showed the highest rate (53%) among patients. Problematic stigma appeared in 24.71% of DM patients. Lower educational level, being divorced or widow, age above 50 years, being unemployed or housewife, and lower income showed significantly higher diabetic stigma scores.

**Conclusion:**

One-quarter of type 2 DM patients showed problematic stigma. The mean diabetic stigma score was significantly higher among patients with lower education, divorced or widow status, older age, unemployment or housewife category, and low-income status.

## Introduction

Diabetes mellitus (DM) is a leading cause of death in low, middle, and high-income countries. Annually about 1.5 million deaths are directly linked to DM. The numbers and rates of diabetes have increased greatly over the last decades. Type 2 DM, which mainly affects adults is the most common type forming nearly 95% of DM cases, and nearly 1 out of 10 adults have diabetes [1–3].

The world prevalence of diabetes among adults is 8.5%. The Middle East and North Africa region have the highest rates of diabetes over other world regions, which nearly double the world figure at 16.1% with an estimated 1 in every 6 adults getting diabetes. The prevalence of diabetes among Iraqi adults is above 13%, and the rates are increasing dramatically [1, 3–7]. A simultaneous increase in the deaths and disability-adjusted life years (DALYs) rates for type 2 DM in the region had been recognized where Iraq is one of the countries that showed a significant increase in the burden of type 2 DM [8, 9].

Patients affected with diabetes need to follow a certain diet and lifestyle modifications and many will take medications probably all through their life. Further, they are prone to many complications and hazards including cardiovascular events, renal insufficiency, visual disturbances, and neurovascular complications. Their quality of life will be impacted by the protracted suffering, and many will encounter additional psychological hazards, including stigma [1, 10–12].

Goffman in the early 1960s described stigma as a sign of social disgrace occurring within public relations there is a negative perception towards a person or people for being beyond ordinary, abnormal, or inferior. Therefore, stigmatized people will be treated differently [11, 13–16].

Further, stigma is categorized into public stigma and self-stigma. The public stigma could be derived from any person or group, coworkers, and might include close relatives or family members who perceive and behave with a person differently, inferiorly, and with discrimination. While self-stigma is a stigma at the individual level that involves accepting general prejudices and preconceptions to diminish oneself. So, the person will suffer from a loss of confidence, lower self-esteem, and self-blame leading to a negative impact on performance and mental health [15, 17–19].

Health-related stigma is referring to stigma linked to grappling with a certain disease or health status, which has been connected with many health conditions including mental illnesses, HIV infection, obesity, and chronic diseases. Diabetic stigma is one of the health-related stigmas, which has been of rising concern in the last two decades where diabetic people are more likely to experience self-stigmatization as well as stigmatization by the community, their relatives, and even health care providers. This stigmatization is likely to worsen the course of the disease, reduce treatment adherence, and affect the psychosocial well-being and quality of life of diabetic patients. The long-term and progressive nature of diabetes exacerbates these issues [12, 20–25]. This study aims to assess the magnitude of stigma related to diabetes mellitus among patients confirmed with type 2 DM.

## Methods

### Study design

An analytic cross-sectional study was performed during 2022, data collection started on 1st June 2022 till 1st November 2022.

### Study population and sampling

All patients aged 18 years and above, diagnosed with type 2 diabetes and visiting the 2 Public diabetic centers in AL-Sader Medical City, AL-Hassan AL-Mujtaba primary health care center (PHCC), and 5 private diabetic clinics in Al-Najaf Governorate.

Patients with acute conditions like severe pain and emergency situations, those not speaking the Arabic language, and those who refused to participate were excluded from the study. Four days a week were assigned for data collection from the selected sites (diabetic center, PHCC, 5 private diabetic clinics). One day for diabetic center, 1 day for PHCC, and 2 days for private clinics. Study participants were recruited through proportional allocation consecutive sampling until fulfilling the required sample size.

The sample size was calculated according to the equation; *n* = *Z*^2^*P*(1 − *P*)/*d*^2^ [26] where n is the sample size, *Z* is the statistic corresponding to 95% confidence (1.96), *P* is the stigma prevalence for T2DM (52%) from a previous study [19], and d is precision (0.05). The minimal sample size required is 421 patients after adding (10% = 38) to compensate for non-response.

### Data collection tool

Data were collected by direct interviews with patients and filling a semi-structured questionnaire derived from a previous study [27] that developed and validated the questionnaire. The Arabic version was requested and downloaded from eprovide website which is available at https://eprovide.mapi-trust.org/ with some modifications, it was tested for validity and reliability by Alzubaidi et al., [28].

The questionnaire consisted of 2 parts; part 1 includes the sociodemographic and diabetes-related questions like age, gender, education, occupation, marital status, place of living, income, duration of DM, prescribed medication, family history of diabetes, perception of lifestyle, perception about diabetic control, and the presence of self-reported DM complications. Part 2 includes 19 questions for the Type 2 Diabetes Stigma Assessment Scale (DSAS-2), all of them were answered on a 5-point Likert scale from strongly disagree to strongly agree. This scale has been classified into 3 subscales including treated differently (6 items), blame and judgment (7 items), and self-stigma (6 items).

The answer choices of each item in the stigma scale were coded as follows: (strongly disagree = 1, disagree = 2, unsure = 3, agree = 4, and strongly agree = 5). The mean stigma score for all items ranges from (19–95), for the treated differently section (items 1, 4, 7, 10, 14, and 17) and self-stigma (6,9,11,13,15,18) is 6–30, and for blame and judgment (items 2, 3, 5, 8, 12, 16, 19) is 7–35. Participants who scored more than one SD above the mean total diabetes stigma score, are supposed to have a potentially problematic stigma for diabetes [27].

### Statistical analysis

Collected data were entered and analyzed by SPSS (statistical packages for social sciences) software program for Windows version 26 [29]. Frequency and percentage were used to describe the categorical variables, while continuous variables were represented by the mean and standard deviation (SD). The difference in means between groups was assessed by independent samples *t*-test and one-way ANOVA test. Multiple linear regression was done to identify the significant predictor variables for stigma perception. A *P* value equal to or less than 0.05 was considered significant.

## Results

Table 1 shows the sociodemographic features of the participant patients. The mean age was 56 years old with about two-thirds (65.5%) being above the age of 50 years. Men represented 265 (61.8%) of the sample. More than half (57.1%) were married. More than half (54.1%) were living in city centers and the majority (83%) lived with their families. Around 63% (269) were considered to have a middle-class monthly income.

**Table 1:** Sociodemographic characteristics of 429 patients with type 2 DM, Al-Najaf Governorate, Iraq, 2022

Variables	Mean	SD
Age (years) (33–84 years old)	56.56	11.59
	*N*	%
Age		
Up to 50 years old	148	34.5
Above 50 years	281	65.5
Gender		
Men	265	61.8
Women	164	38.2
Marital status		
Married	245	57.1
Single	69	16.1
Divorced or widow	115	26.8
Educational level		
No formal education and primary school	145	33.8
Intermediate and secondary school	116	27.0
College and higher education	168	39.2
Job		
Full time	238	55.4
Free employee	71	16.6
Not employed or housewife	120	28.0
Place of living		
City center	232	54.1
District and subdistrict	140	32.6
Village or city side	57	13.3
Living conditions		
With family	356	83.0
Alone	73	17.0
Patient’s opinion about his income		
Low	32	7.5
Middle	269	62.7
Good	128	29.8

In Table 2, data about diabetes history were presented. Most of the patients (77.9%) had a positive family history of diabetes. Regarding the duration of the disease, only 58 (13.5%) have had the disease for less than 1 year, and 53.1% of the patients had the disease for 1 to 5 years. Only 3 (0.7%) were using insulin for their glucose control and the majority (66.4%) used oral antidiabetic drugs. Diabetic complications were reported by 247 patients (57.6%).

**Table 2:** Diabetic history and characteristics of 429 study participants with type 2 DM, Al-Najaf Governorate, Iraq, 2022

Variable	*N*	%
Family history of diabetes		
Present	334	77.9
Absent	95	22.1
Duration of disease		
Less than 1 year	58	13.5
1–5 years	228	53.1
More than 5 years	143	33.4
Lifestyle and dietary	45	10.5
Type of medications		
Oral antidiabetic drugs	285	66.4
Combined	96	22.4
Insulin	3	0.7
Rating of commitment to lifestyle		
No	29	6.8
Medium	300	69.9
Very good	100	23.3
Rating of diabetic control		
Not good	59	13.8
Medium	275	64.1
Good	95	22.1
Presence of diabetic complications		
Yes	247	57.6
No	182	42.4

Type 2 Diabetes Stigma Assessment Scale (DSAS-2) was used to assess the stigma perception among 429 patients previously diagnosed with type 2 diabetes mellitus disease. Responses to scale items were demonstrated in Tables 3, 4 and 5. Each subscale was represented separately. There were 158 (36.9%) patients who agreed and strongly agreed with the feeling that they were treated as sick. While 133(31%) agreed and strongly agreed that they were being rejected by others and 132 (30.8%) agreed that some people think they cannot fulfill their responsibilities. Patients agreed and strongly agreed with the feeling that they were excluded from social events including food and being discriminated against in the workplace with a percentage of 27.2% and 24.9% respectively. Less than one-quarter of the patients (21.2%) thought that some people considered them lesser.

**Table 3:** Responses of patients to the stigma scale (treated differently) about type 2 DM, Al-Najaf Governorate, Iraq, 2022

Subscale	Responses No. (%)				
Treated differently	Strongly disagree No. (%)	Disagree No. (%)	Unsure No. (%)	Agree No. (%)	Strongly agree No. (%)
Some people think I cannot fulfill my responsibilities (e.g., work, family) because I have type 2 diabetes	40(9.3)	243 (56.6)	14 (3.3)	117 (27.3)	15 (3.5)
Some people treat me like I’m “sick” or “ill” because I have type 2 diabetes	25 (5.8)	221 (51.5)	25 (5.8)	141 (32.9)	17 (4.0)
Some people see me as a lesser person because I have type 2 diabetes	45(10.5)	252 (58.7)	41 (9.6)	83(19.3)	8 (1.9)
Some people exclude me from social occasions that involve food/drink they think I shouldn’t have	34 (7.9)	245 (57.1)	33 (7.7)	110 (25.7)	7 (1.6)
I have been discriminated against in the workplace because of my type 2 diabetes	38 (8.9)	243 (56.6)	41 (9.6)	97 (22.6)	10 (2.3)
I have been rejected by others (e.g., friends, colleagues, romantic partners) because of my type 2 diabetes	37(8.6)	239(55.7)	20(4.7)	116(27.0)	17(4.0)

**Table 4:** Responses of patients to the stigma scale (blame and judgment) about type 2 DM, Al-Najaf Governorate, Iraq, 2022

Subscale	Responses No. (%)				
Blame and judgment	Strongly disagree No. (%)	Disagree No. (%)	Unsure No. (%)	Agree No. (%)	Strongly agree No. (%)
I have been told that I brought my type 2 diabetes on myself	17(3.9)	172 (40.1)	27 (6.3)	190 (44.3)	23 (5.4)
There is blame and shame surrounding type 2 diabetes	32 (7.5)	262 (61.1)	31 (7.2)	95 (22.1)	9 (2.1)
Because I have type 2 diabetes, some people assume I must be overweight, or have been in the past	19(4.4)	155 (36.1)	37 (8.6)	171(39.9)	47(11)
Health professionals think that people with type 2 diabetes don’t know how to take care of themselves	21 (4.9)	187(43.6)	29 (6.7)	174 (40.6)	18(4.2)
Because of my type 2 diabetes, health professionals have made negative judgments about me	35 (8.2)	233 (54.3)	36 (8.4)	115 (26.8)	10 (2.3)
There is a negative stigma about type 2 diabetes being a “lifestyle disease”	25(5.8)	220(51.3)	63(14.7)	111(25.9)	10(2.3)
Because I have type 2 diabetes, some people judge me for my food choices	13(3)	157(36.6)	32(7.4)	198(46.2)	29(6.8)

**Table 5:** Responses of patients to the stigma scale (self-stigma) about type 2 DM, Al-Najaf Governorate, Iraq, 2022

Subscale	Responses No. (%)				
Self-stigma	Strongly disagree No. (%)	Disagree No. (%)	Unsure No. (%)	Agree No. (%)	Strongly agree No. (%)
I feel embarrassed in social situations because of my type 2 diabetes	37(8.6)	269 (62.7)	23 (5.4)	93 (21.7)	7 (1.6)
I'm ashamed of having type 2 diabetes	41(9.6)	270 (62.9)	25 (5.8)	87 (20.3)	6 (1.4)
I blame myself for having type 2 diabetes	24 (5.5)	188 (43.8)	28 (6.5)	167 (38.9)	22 (5.1)
Because I have type 2 diabetes, I feel like I am not good enough	32 (7.5)	207 (48.3)	63 (14.7)	111 (25.9)	10 (2.3)
Having type 2 diabetes makes me feel like a failure	44 (10.3)	240 (55.9)	30 (7)	100 (23.3)	15 (3.5)
I feel guilty for having type 2 diabetes	37(8.6)	269(62.7)	23 (5.4)	93(21.7)	7(1.6)

In Table 4, more than half (53%) of patients agreed and strongly agreed that people judge them because of their feeding choices, and around 51% agreed with the people’s assumption that being diabetic is related to being overweight. Nearly half (49.7%) agreed and strongly agreed that they have been told that they brought the disease to themselves. There were 192(44.8%) patients who agreed and strongly agreed with the item: “Health professionals think that people with type 2 diabetes do not know how to take care of themselves”. The above 4 items collected higher percentages of agreement with the stigma scale amongst the whole scale items. Slightly similar percentages (29.1% and 28.2) for those who agreed with the item: healthcare professionals think negatively about them and negative stigma towards diabetes because it is a lifestyle disease. Around one-quarter (24.2%) agreed and strongly agreed that diabetes is related to shame or blame.

Table 5 shows patients’ responses to items related to self-stigma. The highest percentage (44%) agreed and strongly agreed blaming themselves for their disease. Nearly one-third (28.2%) felt they were not good enough because of their diseases. A slightly lower percentage (26.8%) agreed and strongly agreed that diabetes made them feel like a failure. Similarly (23.3%) agreed and strongly agreed that they felt guilty and embarrassed in social situations. Only 21.7% agreed and strongly agreed that they were ashamed of being diabetics.

In Table 6, the descriptive statistics for the stigma scale show the mean total score of the stigma scale was 51.72 ± 16.82 points. For each subscale namely, treated differently 15.50 ± 5.58, blame and judgment 20.15 ± 6.23, and self-stigma 16.05 ± 5.55. The highest mean score percent was 57.57% which belongs to the blame and judgment subscale.

**Table 6:** Descriptive statistics of the stigma scale scoring among the study sample

Descriptive statistics					
Scale score	Minimum	Maximum	Mean	Standard deviation	Mean Score percent
Total scale score	19	94	51.72	16.82	54.47%
Treated differently	6	30	15.50	5.58	51.67%
Blame and judgment	7	35	20.15	6.23	57.57%
Self-stigma	6	30	16.05	5.55	53.50%

**Fig. 1:**
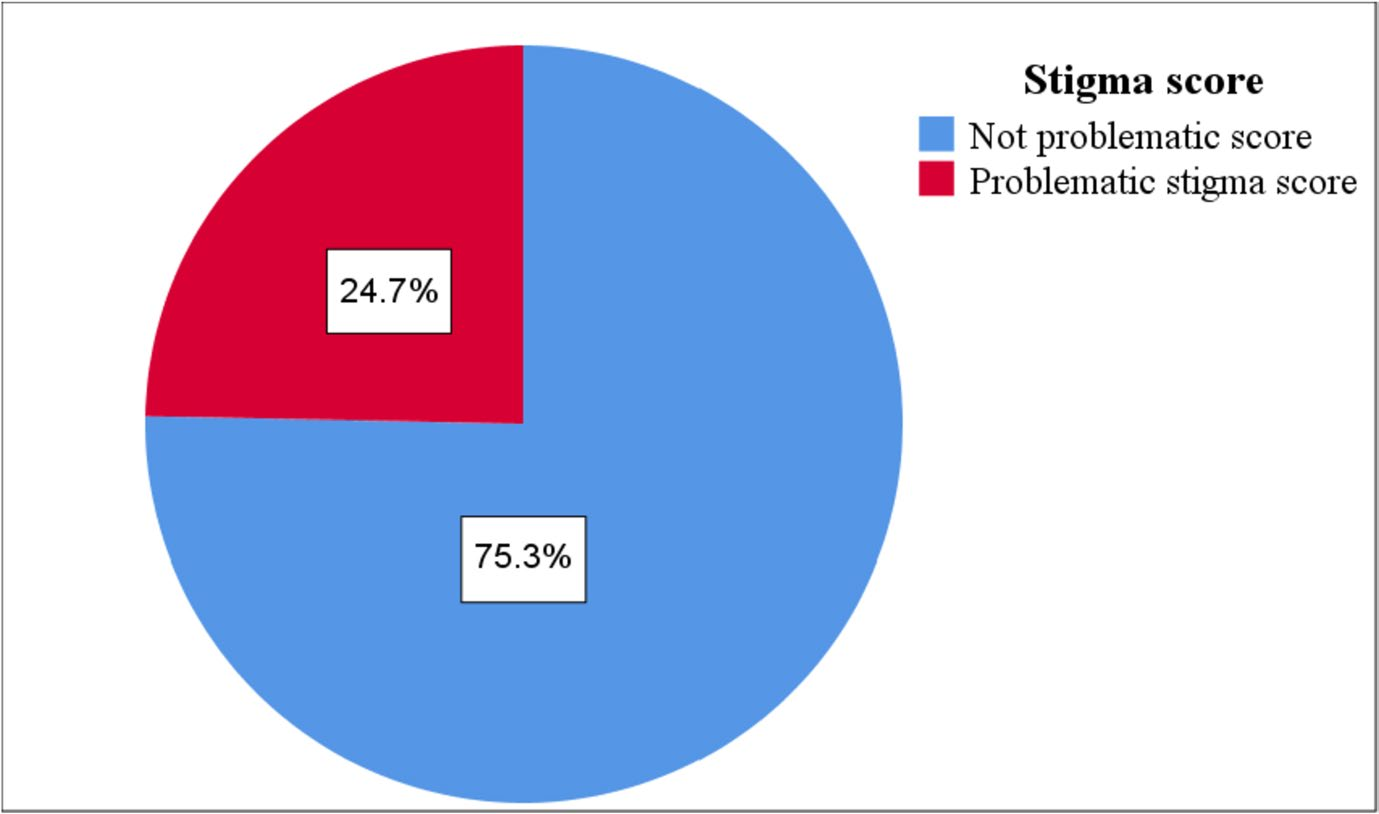
Frequency of patients complaining from potentially problematic stigma score

In Fig. 1, there were 106 (24.71%) patients who are considered to have a problematic stigma score regarding diabetes (those who had a total stigma score more than one SD above the mean, i.e., above 69).

There were significant differences between the mean of the total scale score and subscales with the selected sociodemographic features of patients including age, gender, education, occupation, self-rated monthly family income, marital status, duration of the disease, and place of living with a *P* value less than 0.05 (Table 7). Women had significantly higher means of all stigma subscales than men. Increasing age was also significantly associated with increasing stigma scores among the participants. Patients who were above 50 years old showed significantly higher means for all items of the scale. The result shows that the mean stigma scores were significantly related to the educational level of the included patients. The highest mean of stigma was found in those with lower educational levels. Housewives’ women and those who were not employed were significantly different from patients with full-time employed and free employees. Those who had full-time jobs with lesser stigma scores. A significant difference was seen in mean scores between the patients according to their self-rated income class. Those who perceived being in a lower class demonstrated higher stigma mean scores followed by those in the middle class. Patients who are divorced or widows have a higher stigma means than single or married patients. Patients who lived in city centers showed less mean stigma scale scores than those who lived in the districts and subdistricts. Those who lived in the villages or countryside had significantly higher stigma means scores. Also, those with disease duration above 5 years have significantly higher stigma scores.

**Table 7:** Mean differences in stigma score for the total scale and sub-scales between different sociodemographic variables

Variables	Total scale stigma score	Treated differently	Blame and judgment	Self-stigma
	Mean (SD)	Mean (SD)	Mean (SD)	Mean (SD)
Gender				
Men	50.11(16.05)	14.92(5.29)	19.64(6.07)	15.53(5.31)
Women	54.32(17.75)	16.45(5.91)	20.96(6.41)	16.89(5.85)
*P* value (independent samples *t* test)	0.014	0.007	0.032	0.016
Age				
≤ 50 years	42.44(10.94)	12.31(3.51)	17.08(4.62)	13.04(3.80)
> 50 years	56.60(17.33)	17.18(5.74)	21.76(6.37)	17.64(5.68)
*P* value (independent samples *t* test)	< 0.001	< 0.001	< 0.001	< 0.001
Educational level				
No education and primary	65.82(15.21)	20.20(5.17)	24.82(5.65)	20.80(4.85)
Intermediate, secondary	46.42(12.70)	13.94(4.17)	18.12(4.87)	14.35(4.33)
College and above	43.20(12.24)	12.53(3.89)	17.52(5.19)	13.14(5.55)
*P* value (one-way ANOVA test)	< 0.001	< 0.001	< 0.001	< 0.001
Employment				
Full time	43.62(11.70)	12.88(3.86)	17.35(4.76)	13.37(3.94)
Free employee	53.66(15.76)	15.91(5.17)	21.04(5.92)	16.70(5.27)
Not employed/housewives	66.63(15.53)	20.47(5.27)	25.16(5.69)	20.99(4.96)
*P* value (one-way ANOVA test)	< 0.001	< 0.001	< 0.001	< 0.001
Monthly family income				
Low	72.72 (13.95)	22.87(4.65)	26.81(5.59)	23.30(4.17)
Middle	53.81(16.10)	16.25(5.30)	20.71(6.00)	16.84(5.26)
Good	42.07(12.05)	12.10(3.71)	17.29(5.21)	12.67(4.04)
*P* value (one-way ANOVA test)	< 0.001	< 0.001	< 0.001	< 0.001
Marital status				
Married	47.09 (13.29)	13.91(4.32)	18.59(5.18)	14.57(4.46)
Single	44.23(15.46)	13.20(5.24)	17.55(5.94)	13.47(5.07)
Divorced or widow	66.08(15.93)	20.28(5.37)	25.03(5.82)	16.05(5.55)
*P* value (one-way ANOVA test)	< 0.001	< 0.001	< 0.001	< 0.001
Place of living				
City centers	44.99 (13.26)	13.08(4.27)	18.11(5.35)	13.78(4.47)
District and subdistrict	56.11(16.44)	17.15(5.41)	21.45(6.14)	17.50(5.28)
Village and cityside	68.30(15.82)	21.33(5.05)	25.22(6.10)	21.73(4.98)
*P* value (one-way ANOVA test)	< 0.001	< 0.001	< 0.001	< 0.001
Duration of the disease (years)				
Less than 1 year	40.36(10.73)	11.75(3.24)	16.15(4.92)	12.44(3.52)
1–5 years	47.77(13.88)	14.27(4.70)	18.75(5.24)	14.73(4.62)
More than 5 years	62.62(17.36)	18.99(5.82)	23.99(6.25)	19.63(5.73)
*P* value (one-way ANOVA test)	< 0.001	< 0.001	< 0.001	< 0.001

There was a positive significant correlation between the selected variables with the mean stigma score (*r* = 0.693) and these variables explain 48% of the variance in stigma (*R*^2^ = 0.480, *P* > 0.001). Multiple regression analysis shows that being above 50 years old, having no formal education, unemployed, and being separated from a partner are significant predictors for the stigma scale (Table 8).

**Table 8:** Regression analysis for the main predictors of total stigma score with the selected demographic variables

Variables	*B*	*P* value
Women gender	1.810	0.167
Above 50 years of age	6.984	< 0.001
No formal education or primary school	7.837	< 0.001
Poor subjective income	4.383	0.096
Unemployed or housewife	5.423	0.009
Divorced or widow	7.730	< 0.001
Villages or countryside	1.948	0.365
Duration of disease more than 5 years	2.873	0.074

## Discussion

The stigma among diabetic patients has significant negative consequences on both their metabolic compensation and quality of life [21, 25]. It is significantly associated with higher HbA1C levels, higher body mass index, and poorly controlled blood glucose. Additionally, it affects the emotional aspects of life, which is related to the increased intensity of therapy [19]. This study addressed a research gap as there is limited data about the stigma surrounding type 2 diabetes in Iraq and other Middle East countries.

Current results demonstrated that the mean (SD) for all items' stigma score is 51.72 (16.82), and about a quarter (24.71%) of patients are potentially suffering from a relevant stigma. The means for treated differently, blame, and self-stigma subscales were 15.5, 20.2, and 16.1 respectively. A recent study conducted in Colombia showed that the mean (SD) of DSAS-2 was 49.79 (7.11), and 16.4% of participants are suffering from stigma [22]. An Australian survey of more than a thousand patients reported that the mean (SD) of DSAS-2 was 41.0 (15.9), and more than 19.3% of responders experienced diabetic stigma [27]. Another Australian online survey found that the mean (SD) of total DSAS-2 was 43.5 (16.2), and for those treated differently, blame, and self-stigma subscales were 12.0, 19.2, and 12.3 respectively [30]. Higher stigmatization among our responders could be attributed to the difference in Iraqi culture, as there are multiple factors such as socioeconomic status, educational level, and quality of health services that could play a role in their view of illness in society. Iraqi cultural beliefs such as the perception of people with diabetes were responsible for developing their condition due to misconceptions about the causes of diabetes, such as believing that it is caused by eating sweets or drinking sweetened beverages [31]. Furthermore, Iraqi diabetic patients lack proper knowledge and awareness about diabetes, which could lead to poor self-management practice that is mainly associated with poor glycemic control and diabetic complications which are attributed to higher levels of stigmatization [32].

Social background can influence the stigmatization of diabetes or other chronic diseases as cultural and social contexts mainly shape identities, behaviors, and appearances that are considered appropriate or normal [33]. A similar discrepancy was reported by the World Mental Health Surveys of the perceived stigma associated with mental and chronic physical illnesses in 16 countries, as a higher prevalence of stigma was found in developing in comparison to developed countries (22.1% vs 11.7%) [34].

Nearly half of the patients have been told that they brought diabetes to themselves (49.7%), and they have DM because of their overweight (49.9%), which is higher than previously reported rates (25.7%), and (13.1%) [27], whereas its lower than others (64.1%), and (58.8%), respectively [22].

The highest rate for a single question in the current research with more than half of the participants (53%) agreed and strongly agreed that some people judge them for their food choices because they are having DM. This is greatly higher than the reported rate (9.5%) [30], while it is lower than another (80.1%) [22].

The most commonly described theme of type 2 DM associated with stigma in this study was blaming and judgment mean score percentage equals 57.57%. This is consistent with other studies that found patients always described feeling judged and blamed by others for causing their diabetes through being overweight or obese, or due to inactivity, laziness, poor diet, or overeating [19, 35]. Several studies demonstrated that type 2 DM is a preventable disease [1, 35], emphasizing the role of behavior and personal responsibility in the development of the disease. The increased prevalence of type 2 DM is associated with the development of social stigma. In fact, the role of an individual in the development of type 2 DM may not be obvious immediately, certain risk factors like obesity and the need for daily self-management (e.g. blood glucose checking, modifying diet, and medication taking) may be conspicuous by others and lead to adverse consequences such as stigmatization [36].

Multiple regression analyses of current results demonstrated that older age (> 50 years), lower educational attainment, unemployment, and being widowed or divorced are significantly related to a higher level of stigma (*P* < 0.05). Other researchers also found that sociodemographic variables were related to diabetic stigma; they reported higher stigmatization with younger age [16, 21, 25], and lower educational attainment [25]. In contrast, Pedro et al. reported that age was not associated with diabetic stigma [22]. Whereas Kato et al. reported that patients who had not announced their diabetes status tended to be older, have lower educational levels, and be employed part-time [37]. These contradictories in the findings could be attributed to differences in the sample size, culture, and the distribution of sociodemographic characteristics of studied populations. Age is linked to a greater increase in stigma in the situation of increased limitation and greater functional limitation. Older people often experience stigma related to aging. They might suffer from double stigma if they are having other health problems, in addition to negative behavior and attitudes against the elderly [38, 39].

A lower educational status was strongly associated with negative physical and mental outcomes [40]. Low-educated people are less knowledgeable about their illness, and this may lead to the expression of a higher level of stigma. It is also possible that those of lower education have lower access to healthcare services. Thus, they would probably have been associated with poor outcomes such as amputations or retinopathy which affect functioning and may lead to stigma.

In fact, in diabetic patient care, there is a need for continuous medical review and financial support, especially in patients with multiple chronic illnesses [19]. Unemployed and housewives usually suffer from higher stigmatization as they are economically dependent on others.

Additionally, diabetic patients suffer from a higher level of stigma due to lower social support which reduces their ability to disease management [41]. Therefore, divorced, and single person has suffered from a higher level of diabetic stigma probably due to a lack of spousal support in the management of their illness.

### Limitations of the study

A limitation of this study is that clinical data regarding diabetes control, complications, and commitment to the lifestyle were self-reported by the responders which is subjective, the same issue applied to economic status**.** However, to our knowledge, this is the first study in Iraq that investigated the stigma of type 2 DM.

## Conclusion

Stigma with type 2 diabetes mellitus is a considerable problem. Older age, lower educational attainment, divorced or widow, and unemployment are socio-demographic factors that are associated with higher stigmatization. Implementation of an educational program for the health care workers, family members, and friends of diabetic patients is recommended as an intervention for stigma reduction.

## Data Availability

The data are available upon request from the corresponding author.

## Abbreviations

DM: Diabetes mellitus
DSAS: Diabetes Stigma Assessment Scale
T2DM: Type 2 Diabetes mellitus
SPSS: Statistical packages for social sciences
SD: Standard deviation
SME: Self-management education
